# Factors associated with birthweight decline in Japan (1980–2004)

**DOI:** 10.1186/s12884-021-03819-0

**Published:** 2021-04-27

**Authors:** Noriko Kato, Catherine Sauvaget, Honami Yoshida, Tetsuji Yokoyama, Nobuo Yoshiike

**Affiliations:** 1 Department of Early Child Care and Education, Jumonji University, Niiza, Japan; 2 Department of Epidemiology, National Institute of Public Health, Wako, Japan; 3 Center for Innovation Policy, Kanagawa University of Human Services, Yokosuka, Japan; 4 Department of Health Promotion, National Institute of Public Health, Wako, Japan; 5 Department of Nutrition, Aomori University of Health and Welfare, Aomori, Japan

**Keywords:** Birthweight, Gestational age, Maternal body mass index, Japan

## Abstract

**Background:**

Birthweight has been declining consistently for more than 30 years in Japan. This rapid rise in low birthweight is one of the worst among the countries of the Organization for Economic Co-operation and Development. We examined potential determinants of birthweight decline in Japan.

**Methods:**

We used population-based birth certificate data from vital statistics records and retrieved 40,968,266 birth certificates of neonates born between 1980 and 2004. We analyzed time trends using linear regression analysis in examining whether the decline in birthweight could be explained by obstetrical variables such as gestational age and multiple gestations.

**Results:**

From 1980 to 2004, we observed a decline in mean birthweight with a yearly effect of − 8.07 g, which became steeper after 1985, persisted until 1999, and plateaued thereafter. After adjusting for gestational age, gender, birth order, multiple gestations, and fathers’ age, the yearly effect between 1980 and 2004 persisted at − 5.13 g.

**Conclusion:**

Recent decreases in birthweight among Japanese neonates were not fully explained by trends in gestational age, gender, birth order, multiple gestations, and fathers’ age. Thus, additional factors such as pre-pregnancy maternal body mass index (BMI) and maternal diet should be considered. Reducing the rate of induced deliveries and improving the BMI or diet of young women should be a public health priority.

## Background

Birthweight has been declining consistently for more than 30 years in Japan [1]. The mean birthweight in Japan is generally low; it was 3000 g (3050 g for males and 2960 g for females) in 2018 [1]. Comparatively, in many countries, the mean birthweight values are higher. For instance, the mean birthweight in England and Wales was 3316 g (2012) [2], 3322 g in Canada (2018) [3] and 3200 g in the Republic of Korea (2016) [4].

Concomitantly with this declining birthweight, the proportion of infants with low birth weight, defined as birthweight less than 2500 g, is high and has been increasing [5] rapidly in Japan. The proportion of low birthweight infants in 1990 and 2015 was 6.3 and 9.4%, respectively [6], while that in average of the Organization for Economic Co-operation and Development (OECD) countries was 5.7 and 6.5% [7]. Due to the rapid increase in the number of low birthweight infants in Japan, it has been ranked the fifth worst country among the OECD countries [7]. Many countries have also experienced a decline in birthweight in the past few years, including the US [8], Scandinavian countries [9], Spain [10] and the Republic of Korea [4], but this is not as rapid as that in Japan.

Factors associated to low birthweight include short gestational age [11, 12], maternal smoking habit [13, 14], low pre-pregnancy maternal body mass index (BMI) [15], low gestational weight gain [15], anemia [16], and low socioeconomic status [17] among others. Studies from Japan reported additional factors of low birthweight, including high maternal age (more than 30 years of age), low BMI (less than 18.5 kg/m^2^) [5, 18], and preterm birth [5, 11].

Birth weight decline in Japan began in 1975 and continued for 30 years. In 2000, the Ministry of Health, Labour and Welfare (MHLW) promulgated a national campaign named Healthy Parents and Children 21, in which improvement of birth weight was an important target of the campaign. Meanwhile, in 2006, MHLW established guidelines regarding optimal gestational weight gain of expectant mothers based on pre-pregnancy BMI to guarantee that neonates have an adequate and healthy birthweight of approximately 3000 g [12]. Additionally, pregnant women were encouraged to consume sufficient nutrients.

In the final assessment of the first stage of the Healthy Parents and Children 21 campaign in 2014, birthweight decline had not recovered, indicating that low birthweight is a persistent and severe issue. For the second phase, which began in 2015, the improvement of birthweight is remaining a priority. To contribute to the campaign, it is necessary to clarify the modifiable factors affecting birthweight. Hence, we aimed to examine the period in which the birthweight rapidly decreased and clarify factors contributing to the marked decline.

## Methods

### Data source

Exhaustive national database such as birth certificates were used in the analyses. Since birth certificates are gathered from all births occurring in Japan under the Family Register Act and include data on birthweight and other demographic information, we used this information to examine the potential determinants of the national birthweight decline.

In Japan, birth certificates are stored systematically by the MHLW on electronic data files. These certificates are filled by obstetricians or midwives following obstetric recordings in the hospitals or clinics and are filed in the municipalities’ health departments into the MHLW database. This database is anonymous and includes information related to neonates’ gender, birthweight, birth length, gestational age, multiple gestation, parity, fathers’ and mothers’ age, and birthplace.

**Fig. 1:**
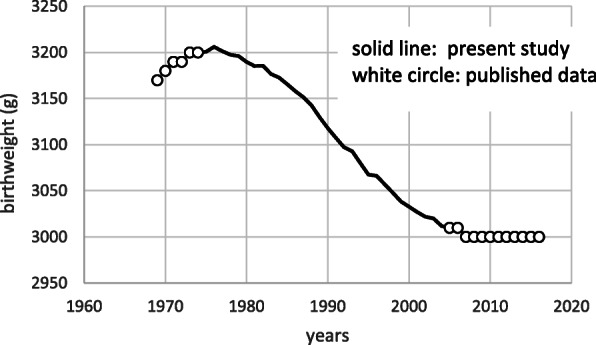
Secular trend of mean birthweight. Published data is from Annual report of vital statistics of Japan (reference [1])

With permission from the Statistics and Information Department, Minister’s Secretariat, MHLW, a total of 40,968,279 birth certificate files were retrieved between 1980 and 2004. Before filling in the birth certification form, parents were informed about privacy protection and the use of public welfare data. National registration of vital records such as birth certificates is exhaustive, based on the Law. Since this study was considered of public interest, it was approved by the MHLW. We used the data from this specific time frame (1980–2004) because the mean birthweight showed an apparent decline during this period (Fig. 1).

### Dependent variable (birthweight)

From 1980 to 1994, birthweights were recorded to the nearest 100 g, which did not exceed the actual birthweight value. For example, if the measured birthweight was 3126 g, it was recorded as 3100 g. Similarly, gestational age was recorded as completed weeks; for instance, if the actual gestational age was 38 weeks and 3 days, it was recorded as 38 weeks. To estimate the most probable value assuming that the distribution was uniform through 100-g intervals or 1-week intervals, 50 g were added to each recorded birthweight value and 0.5 week was added to each recorded gestational age values.

From 1995 to 2004, birthweight was recorded in actual grams; hence, the recorded unit values were used for analysis. In the same period, gestational age was recorded as completed weeks and additional days. For linear regression analysis, the gestational age values were converted into weeks; for example, 38 weeks and 3 days was converted into 38 and 3/7 weeks and entered as 38.429 weeks; the values were expressed as whole numbers and decimals for the analysis.

### Independent variables

The following variables, considered as potential determinants of birthweight, were collected from the birth certificates: gestational age, father’s age, mother’s age, delivery rank, number of the fetuses (singleton, multiple), live birth number, newborn gender, year of birth, prefecture code.

The exact values for father’s and mother’s age, prefectural number, and delivery number codes were reported. For gestation codes, 1 stands for singletons and 2 stands for multiple births. For gender, 1 stands for male and 2 stands for female.

### Statistical analysis

First, we calculated Pearson’s correlation coefficients between birthweight and independent variable. Then, we calculated means and standard deviations of the variables in 1980 (the beginning of the observation) and in 2004 (the end of the observation).

Low birth weight and preterm delivery are both strongly associated with birthweight declines. Therefore we stratified the independent variables into low birthweight or non-low birthweight groups (less than 2500 g; 2500 g and over), and preterm delivery or non-preterm delivery groups (less than 37 weeks gestation; 37 weeks or more). The mean value of each independent variable and its standard deviation were calculated for the two birth weight categories and the two term categories, for the years 1980 and 2004.

To clarify the factors affecting birthweight decline, we used the multiple linear regression analysis [12, 19] for all births from 1980 to 2004.

The effect of time on birthweight was analyzed for each year, first without adjustment (crude effect), then in adjusting for explanatory variables added one by one in the model. We analyzed through total period (1980–2004) and among five-year periods (1980–1984, 1985–1989, 1990–1994, 1995–1999 and 2000–2004). A *P*-value of less than 0.05 was considered statistically significant. All analyses were performed using SAS version 9.1 (SAS Institute, Cary, NC, USA).

The study protocol was approved by the Ethics Committee of the National Institute of Public Health. This study was conducted in accordance with the ethical principles established by the Declaration of Helsinki, 2008.

## Results

From 1980 to 2004, we observed a decrease in birthweight, which became steeper after 1985, persisted until 1999, and slowed down thereafter (Fig. 1).

Gestational age, mother’s and father’s mean age, delivery related variables, neonate gender, place of delivery were statistically associated with birthweight; but the strongest association was observed with gestational age (Table 1). All these variables except for mother’s age and prefecture number were included in the regression analysis. Table 1 also reports that, as compared to 1980, in 2014 the gestational mean age decreased, the mother’s and father’s mean age increased, the mean number of deliveries decreased, as well as the mean number of live births; while the gender distribution and multiple gestation remained similar.

**Table 1:** Correlation of variables with birthweight (1980–2004) and change in variables between 1980 and 2004

variables	units	correlation with birthweight		1980 (*n* = 1,569,777)		2004 (n = 1,110,721)	
		r^2^	*p*-value	mean	SD	mean	SD
Birthweight	grams			3190	444	3014	441
Gestational age	weeks	0.2787	< 0.0001	39.25	1.66	38.86	2.10
Father age	years of age	0.0007	< 0.0001	30.33	4.21	31.68	5.62
Mother age	years of age	0.0003	< 0.0001	27.62	3.75	29.69	4.72
Delivery number	including present birth	0.0084	< 0.0001	1.80	0.84	1.70	0.82
Multiple gestation	1 = singleton 2 = multiple birth	0.05	< 0.0001	1.02	0.13	1.02	0.15
live birth number	including present birth	0.0078	< 0.0001	1.78	0.83	1.69	0.81
Neonatal gender	1 = male 2 = female	0.0086	< 0.0001	1.49	0.50	1.49	0.50
Year	A.D.	0.0183	< 0.0001				
Prefecture number	1 to 47	0.0004	< 0.0001				

The mean values of the independent variables were also calculated for 1980 and 2004, after stratification into low birth weight and non-low birth weight (Table 2), and after stratification into preterm delivery and non-preterm delivery (Table 3). Gestational age was lower in the low birthweight group as compared to the non-low birthweight group; the mean values remained similar between 1980 and 2004. Father’s and mother’s mean ages increased with time. Multiple gestation was more frequent (higher mean value of gestation number) among the low birth weight group and the preterm group in 2004, as compared to 1980.

**Table 2:** Comparison between LBW and non-LBW neonates in 1980 and 2004

variables	units	1980				2004			
		LBW (*n* = 81,665)		non-LBW (*n* = 1,488,112)		LBW (*n* = 104,842)		non-LBW (*n* = 1,005,130)	
		mean	SD	mean	SD	mean	SD	mean	SD
Birthweight	grams	2156	366	3246	372	2158	396	3103	338
Gestational age	weeks	36.40	3.43	39.41	1.34	36.28	3.07	39.10	1.24
Father age	years of age	30.34	4.61	30.33	4.19	32.07	5.81	31.64	5.60
Mother age	years of age	27.69	4.24	27.62	3.72	30.09	4.89	29.65	4.69
Delivery number	including present birth	1.78	0.94	1.80	0.84	1.69	0.86	1.70	0.81
Multiple gestation	1 = singleton 2 = multiple birth	1.03	0.16	1.00	0.07	1.17	0.38	1.01	0.08
Live birth number	including present birth	1.75	0.91	1.78	0.82	1.68	0.85	1.69	0.81
Neonatal gender	1 = male 2 = female	1.53	0.50	1.48	0.50	1.54	0.50	1.48	0.50

**Table 3:** Comparison between preterm and non-preterm neonates in 1980 and 2004

variables	units	1980				2004			
		Preterm (*n* = 63,821)		non-Preterm (*n* = 1,505,946)		Preterm (*n* = 62,935)		non-Preterm (*n* = 1,047,338)	
		mean	SD	mean	SD	mean	SD	mean	SD
Birthweight	grams	2396	608	3223	402	2191	590	3063	377
Gestational age	weeks	34.27	2.46	39.47	1.23	34.28	2.73	39.11	1.17
Father age	years of age	30.70	4.78	30.31	4.19	32.49	5.95	31.63	5.59
Mother age	years of age	28.10	4.37	27.60	3.71	30.43	5.00	29.65	4.69
Delivery number	including present birth	1.93	1.01	1.79	0.83	1.82	0.92	1.69	0.81
Multiple gestation	1 = singleton 2 = multiple birth	1.03	0.18	1.01	0.09	1.22	0.41	1.01	0.10
live birth number		1.89	0.98	1.78	0.82	1.80	0.91	1.68	0.80
Neonatal gender	1 = male 2 = female	1.43	0.50	1.49	0.50	1.44	0.50	1.49	0.50

As shown in Table 4, from 1980 to 2004, we observed a decrease in birthweight with a yearly effect of − 8.07 g. After adjustment for gestational age, birthweight decreased annually by 5.63 g. For further adjustment, explanatory variables were added subsequently. After adjustment for gestational age and neonatal gender, birthweight decreased yearly by 5.60 g. After adjusting for all variables, the decrease weakened to 5.13 g/year. In the early period between 1980 and 1984, the decrease in birthweight was relatively small. The crude effect was − 3.98 g/year, and it became − 0.07 g/year after adjustment by gestational age and − 0.53 g/year after adjustment for all variables. In 1985–1989, where birthweight decline began to become steep, crude yearly effect was − 8.50 g/year, after adjustment for gestational age: − 3.60 g/year and − 3.66 g/year after adjustment for all variables. During the two periods 1990–1994 and 1995–1999, the reduction in birthweight was the steepest within the study period. The crude yearly effect was − 8.71 and − 7.66 g/year respectively, which became − 7.58 and − 7.01 g/year after adjustment for gestational age and became − 5.99 and − 6.26 g/year after adjustment for all variables. Lastly, between 2000 and 2004, the decrease in birthweight declined. The crude yearly effect was − 4.84 g/year, which reduced to − 3.04 g/year after gestational age adjustment and to − 2.87 g/year by adjustment for all variables.

**Table 4:** Multivariate analysis with sequential adjustment, according to several time periods

**All births**	1980–1984 (early period)			1985–1989			1990–1994		
Yearly effect	Birth weight (g)	Standard error	r^2^	Birth weight (g)	Standard error	r^2^	Birth weight (g)	Standard error	r^2^
Crude	−3.98	0.11	0.0002	−8.50	0.12	0.0007	−8.71	0.13	0.0008
Adjusted for gestational age	−0.07	0.10	0.2151	−3.60	0.10	0.2483	−7.58	0.11	0.2843
Plus neonatal gender	0.02	0.10	0.2289	−3.53	0.10	0.2628	−7.57	0.10	0.2991
Plus delivery number	−0.19	0.10	0.2441	−3.42	0.10	0.2805	−6.32	0.10	0.3184
Plus multiple gestation	−0.32	0.10	0.2607	−3.42	0.10	0.2948	−5.97	0.10	0.3326
Plus live birth number	−0.33	0.10	0.2607	−3.47	0.10	0.2948	−5.97	0.10	0.3226
Plus father age	−0.53	0.10	0.2592	−3.66	0.10	0.2936	−5.99	0.10	0.3316
**All births**	1995–1999			2000–2004 (late period)			1980–2004 (total period)		
Yearly effect	Birth weight (g)	Standard error	r^2^	Birth weight (g)	Standard error	r^2^	Birth weight (g)	Standard error	r^2^
Crude	−7.66	0.13	0.0006	−4.84	0.13	0.0002	−8.07	0.01	0.0175
Adjusted for gestational age	−7.01	0.10	0.3150	−3.04	0.10	0.3456	−5.63	0.01	0.2872
Plus neonatal gender	−7.02	0.10	0.3302	−2.99	0.10	0.3607	−5.60	0.01	0.3015
Plus delivery number	−6.42	0.10	0.3480	−2.96	0.10	0.3754	−5.16	0.01	0.3180
Plus multiple gestation	−6.28	0.10	0.3621	−2.79	0.10	0.3884	−5.06	0.01	0.3327
Plus live birth number	−6.28	0.10	0.3621	− 2.79	0.10	0.3884	−5.07	0.01	0.3327
Plus father age	−6.26	0.10	0.3613	−2.87	0.10	0.3872	−5.13	0.01	0.3315

## Discussion

From 1980 to 2004, the mean birthweight decreased in Japan, specifically between 1990 and 1999. Although 2004 was 17 years ago, the birthweight trend was almost constant after 2004. Using data of years in which the birthweight decline was rapid would provide further insight into possible factors contributing to the decline in low birthweight through multiple regression analysis. However, based on our analyses, the decline was not fully explained after adjusting for variables obtained from the birth certificates.

If the birthweight decline was fully explained by the explanatory variables adjusted year effect would get close to zero, like during the period 1980–1984. However, attenuation was only observed partly during the total year period (1980–2004), and during the steepest periods (1985–1989, 1990–1994).

As expected, the birthweight decline was mainly associated with gestational age. After adjusting for all variables, the decrease in birthweight was 5.13 g/year, and adjusting for gestational age solely 5.63 g/year decrease, compared to a crude reduction of 8.07 g/year. This demonstrates that gestational age is not the only factor responsible for the entire birthweight decline.

The fact that birthweight decline was not fully explained by the variable in the regression analysis suggests that other factors than those included in the regression analysis played some role. Especially in the specific period where birthweight decline was rapid, the effect of adjustment for all variables was limited, and gestational age effect was also relatively small. This means that other determinants are associated with rapid birth weight decrease.

Similarly, based on data from the US [12], gestational age partially explained birthweight decline in the regression analysis. The crude yearly effect between 1990 and 2004 was − 3.0 g/year, while it counted − 1.9 g/year after adjustment by gestational age. The fact that birthweight decline was not fully explained by gestational age was consistent with the work by Morisaki et al. [20], where birthweight decline appeared in all gestational age subgroups in the US datasets.

The strength of the present study comes from the large, nationally representative dataset that would not change for decades and from the use of information on potential factors associated with trends in birthweight. The limitations of our study come from the lack of detailed information such as comparing birthweight decline among subgroups classified by gestational age, fetal growth and mode of delivery [12].

Indeed, one of the limitations of this study was gestational age estimation. Gestational age was entered in the birth certificate by doctors or midwives. Some used the date of the last normal menstrual period, while others the age estimate based on the early fetal ultrasonography results. This might have introduced variability and affected the validity of our gestational age estimation. Schonberg et al. [21] determined that gestational age calculated from the last menstrual period is reasonably accurate among term births.

Many factors affect birthweight; however, the following factors could not be analyzed in the present study: medical conditions during pregnancy, childbirth, and other modifiable factors, such as pregnancy diabetes, pregnancy hypertension, placental abruption and abnormal obstetric bleeding. Considering obstetrical practice, gestational age is affected by the mode of delivery. The induction of labor has also steadily increased in Japan [22]. Based on the national growth survey, the rates of cesarean deliveries have increased from 19% in 2000 up to 25% in 2010 [23] and to 25.8% in 2017 [24]. These factors might cause a decline in gestational age. Data from the US showed that a decrease in gestational age was associated with an increase in the number of induced labor [8].

Another limitation of this study is the lack of data likely associated with birthweight, such as maternal smoking status, pre-pregnancy weight, and maternal diet during pregnancy. Our data did not provide information on pre-pregnancy medical conditions such as diabetes and hypertension, which affect birthweight by causing large gestational age neonates and fetal growth restrictions, leading to low birthweight. Maternal smoking restricts fetal growth and increases obstetrical complications and the risk of preterm birth; these factors are likely to induce low birthweight and even stillbirths [13]. In Japan, the smoking prevalence among pregnant women was 5.0, 10.0, 5.0 and 4.9% in 1990, 2000, 2010 and 2013, respectively [25, 26]. Moreover, in a recent survey on mothers and children aged 3–4 months, low birthweight was significantly associated with maternal smoking [14]. A decrease in the proportion of pregnant women who smoked could be a cause of decrease in low birthweight after 2000. In Japan, the Health Promotion Law was enforced in 2002, and individuals were encouraged to quit smoking thereafter. In Canada, intervention studies on pregnant women including smoking cessation, decreased the proportion of low birthweight [27].

**Fig. 2:**
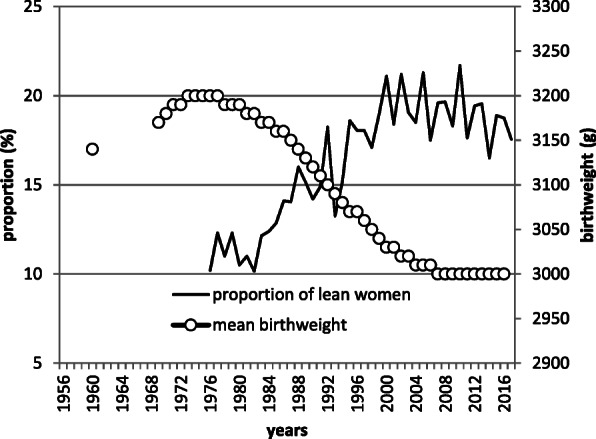
Secular trends of proportion of lean women and mean birthweight. Mean birthweight is from Annual report of vital statistics of Japan (reference [1]). Proportion lean woman means proportion of woman under BMI of 18.5 aged between 20 to 39 years, which are calculated from Annual report of health and nutrition (reference [25])

**Fig. 3:**
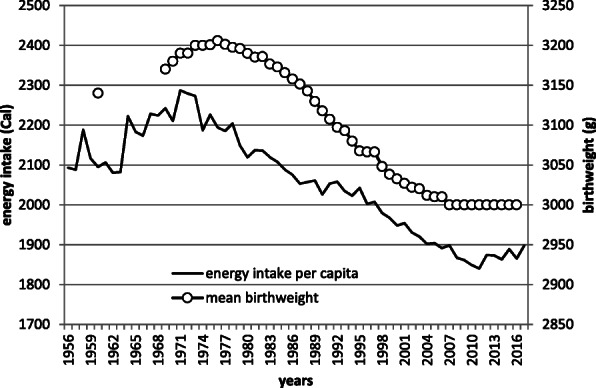
Secular trends of calorie intake per capita (both male and female) and mean birthweight. Mean birthweight is from Annual report of vital statistics of Japan (reference [1]). Calorie intake per capita is from Annual report of health and nutrition (reference [25])

Pre-pregnancy BMI and gestational weight gain are other important factors that affect birthweight [15]. BMI distribution among women within reproductive age could be a proxy for pre-pregnant BMI. The prevalence of underweight has been increasing over the decades in Japan, contrasting with other countries [28]. According to the National Health and Nutrition Survey [29], the proportion of women with a BMI of less than 18.5 kg/m^2^ in the age group of 20–39 years is increasing concomitantly with a decline in the mean birthweight (Fig. 2). For the causality between maternal BMI and birthweight, further studies are needed. As for the effect of maternal diet, the Japanese National Health and Nutrition Survey has revealed synchronized time trends of birthweight and per capita calorie intake (Fig. 3); however, the causality is still unclear. Time-trend synchronization was suggested between birthweight and BMI in women of reproductive age and birthweight, and energy intake of the whole population. The decline in energy intake of the entire population is a reflection of the decrease in macronutrient intake in pregnant women, which is likely to correlate with low birth weight. These factors other than gestational age can most likely result in low birthweight and their effect may be more substantial than that of gestational age.

An increase in maternal age [30] has been shown to be a factor associated with decreased birthweight in the Republic of Korea. In our study, maternal age did not show a linear correlation with birthweight and was not included as an explanatory variable. Another maternal condition that lowers birthweight is anemia and parasitic infections. In Pakistan, the relationship between iron-deficiency anemia and low birthweight was identified [16], and in Sudan [31], the effects of maternal malaria on low birthweight have been reported. Among Japanese mothers living in urban area, hemoglobin change during pregnancy was inversely associated with birthweight [32].

**Fig. 4:**
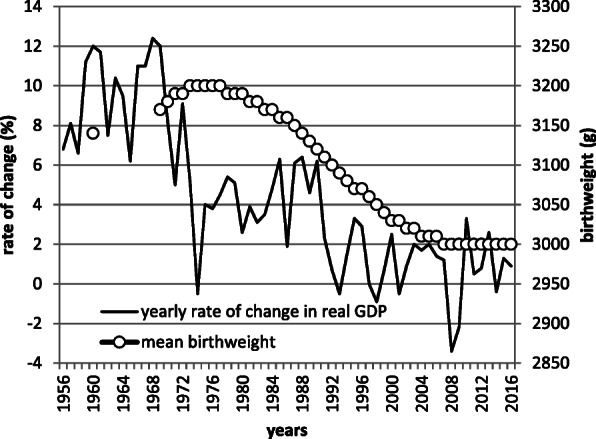
Secular trends of yearly change in GDP and mean birthweight. Mean birthweight is from Annual report of vital statistics of Japan (reference [1]). Yearly change in GDP is from System of national accounts (reference [32–34])

Although socioeconomic status is a well-known factor affecting birthweight [17], birth certificates do not contain any variable which allow analysis related to this factor. A comparison of secular trends of economic growth [33–35] and mean birthweight in Japan is shown in Fig. 4. While the periods of deterioration in economic growth and that of birthweight are almost the same, the association is unclear. Yorifuji et al. [36] pointed out that socioeconomic position is related to air pollution, which influences the occurrence of low birthweight in Japan, suggesting the importance of socioeconomic factors. Further, climate affects birth outcomes; in a study including 32 million US singletons [37], extremely high temperature was associated with preterm birth, which has a strong correlation with low birthweight. In the Japanese setting, urbanization has caused long-term climate changes related to a temperature rise [38]. Thus, such factors should also be considered.

It is well-known that birth size influences not only short-term conditions but also long-term prognosis [39]. Factors lowering birthweight, although not analyzed fully in the present study, could cause various health problems among children as they grow up and even in their adult lives. Indeed, the incidence of low birthweight has an important public health impact because of its association with developmental delay, and longer hospital stay in neonates associated with an increased burden of healthcare costs. In adult life, low birthweight predisposes individuals to an increased risk of metabolic syndrome, chronic diseases and increased mortality [39]. Therefore, a follow-up study is necessary to investigate which sequalae would derive from low birthweight neonates. The findings of present study will not only contribute health policy to improve birth weight and recover from birthweight decline, but also be suggestive for other countries where birthweight decline is on-going.

In conclusion, our study based on data from birth certificates showed that infants’ birthweight has decreased over the decades. These findings might partially be explained by the decline in gestational age, considered to result from a change in the delivery mode. Thus, further studies are needed to determine the clinical and social significance of these findings.

## Data Availability

The data were provided by the Statistics and Information Department of the Minister’s Secretariat at the Ministry of Health, Labor, and Welfare with permission from the Ministry. The data could be obtained if claimed and permitted.

## Abbreviations

OECD: Organization for Economic Co-operation and Development
MHLW: Ministry of Health, Labour and Welfare
